# Remote sensing mapping of structural and hydrothermal alteration in the mougueur inlier, Eastern high atlas, Morocco

**DOI:** 10.1038/s41598-025-99402-0

**Published:** 2025-04-29

**Authors:** Tarik Amraoui, Hassan Ibouh, Abdelouahed Farah, Youssef Bammou, Ali Shebl

**Affiliations:** 1 Laboratory of Georessources, Geoenvironment and Civil Engineering, Department of Earth Sciences, Faculty of Sciences and Technologies of Marrakesh, Cadi Ayyad University, 40000 Marrakesh, Morocco; 2 GEOANLYSIS Morocco, Engineering Consulting Office of Geological Geophysical, and Environmental Services, APPT 4, 2nd Floor, Building 52, Lot Aazouzia, Industrial District, Marrakech-Menara, Morocco; 3 Department of Mineralogy and Geology, University of Debrecen, Debrecen, 4032 Hungary; 4 Department of Geology, Tanta University, Tanta, 31527 Egypt

**Keywords:** Remote sensing, Mougueur inlier, Mining prospecting, Multi-criteria analysis, Eastern high atlas Morocco, Geology, Mineralogy, Petrology

## Abstract

Located north of the town of Gouramma, the Mougueur inlier in the eastern High Atlas of Morocco a part of the Hercynian chain of the Paleozoic Era. It is known for its richness in carbonate vein mineralization (e.g., siderite, ankerite) and Zn–Pb (± Fe, Cu, and Mg) association. The current research integrated remote sensing datasets (e.g., ASTER and Landsat OLI), field observations and petrographic investigations to explore structural and hydrothermal alteration zones linked to the mineralization. Our results revealed that the key tectonic structures trend NE to E–W, control the distribution of mineralization in the Mougueur Inleir and meso-cenozoic cover. Hydrothermal alteration mapping identified abundant iron-bearing minerals and quartz, closely associated with fault zones sush as Tit N’Ali, Tijane, Talharit, and Tamelahl. A spatial overlay analysis of alteration indices, lineaments, geological field surveys shows that anomaly zones correlate strongly with known mineralized structures, suggesting that mineralization is primarily structurally controlled. This integrated methodology demonstrates the power of remote sensing techniques for identifying potential exploration targets and offers a promising tool for future mineral prospecting in similar terrains.

## Introduction

Ongoing, extensive mineral deposit exploration is imperative due to the increasing global population and rising mineral consumption, necessitating access to new mineral resources. Given the availability of extensive datasets and advanced technologies, this is now more achievable. For instance, remote sensing datasets emerge as valuable tools in mineral exploration, enabling strategic exploration over vast regions and thereby reducing exploration time and costs^1,2^. Several studies have utilized different remote sensing datasets for various geological applications. These include the use of multispectral^3,4^ and hyperspectral data^5–7^ for tasks such as lithological mapping. In addition, remote sensing has been effectively applied for hydrothermal alteration mapping^8–11^ and structural analysis^12,13^. Researchers have also employed satellite data for lineament extraction^12–16^ and textural analysis^4^. Moreover, satellite imagery has proven successful in identifying mineral deposits^17–19^, highlighting its value in mineralization exploration.

The Eastern High Atlas (EHA) is a mountainous region of great geological complexity, known for its significant mineral resources^14,20^. This area extends over several tens of kilometers and is distinguished by its varied geology, shaped by tectonic and sedimentary processes that occurred during the Paleozoic, Mesozoic and Cenozoic eras^21^. These processes have favored the formation of various mineral deposits making the EHA an area of interest for mining. The deposits of the EHA mainly consist of metals of great economic value, such as lead, zinc, copper and silver^22^. These metals often occur in the form of veins or stratiform masses, associated with sedimentary and volcanic formations (Table 1). Hydrothermal processes, which enriched these rocks, are responsible for the concentration of these metals, making certain deposits particularly rich and attractive for exploitation.

**Fig. 1:**
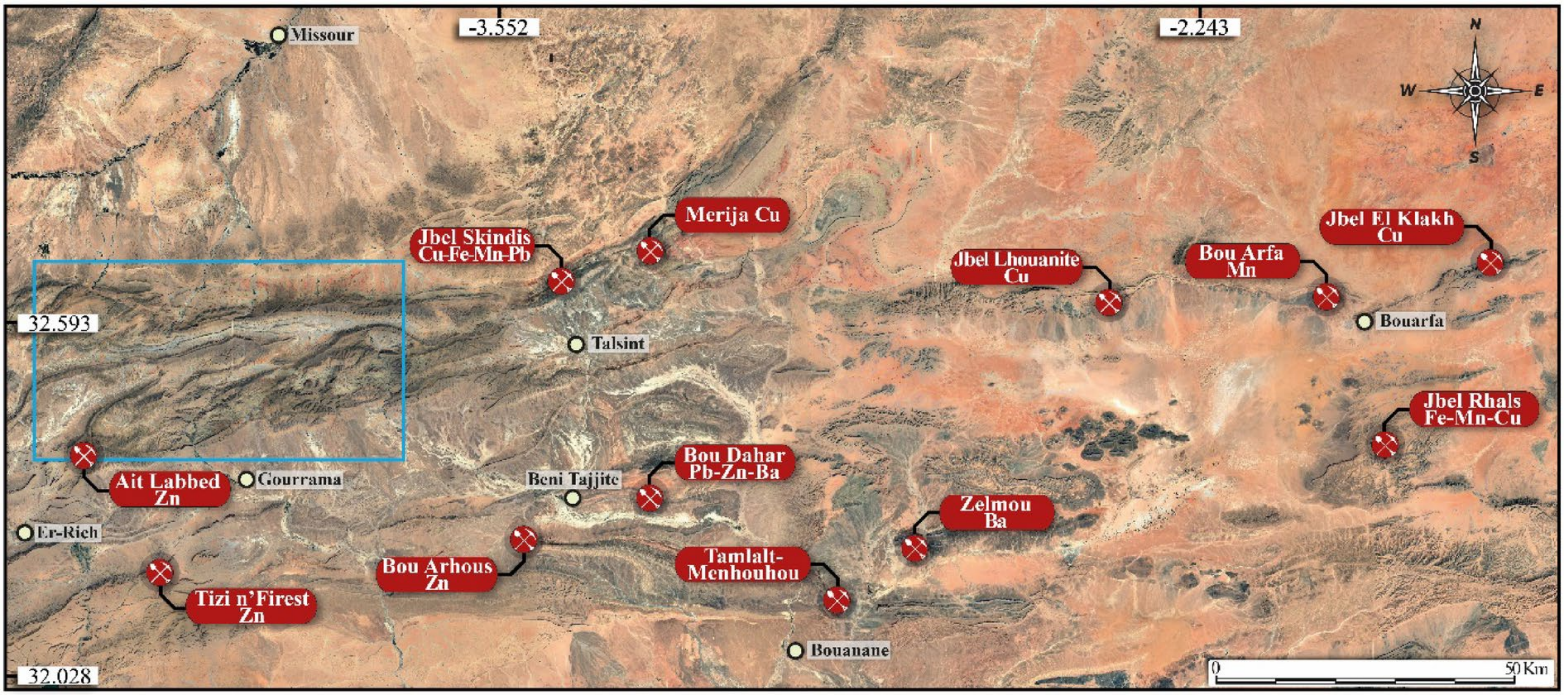
Main deposits of the Eastern High Atlas. The blue rectangle shows the borders of the study area. Created by ArcGIS Desktop 10.8. https://www.esri.com/en-us/arcgis/products/arcgis-desktop/overview.

The history of exploration and mining in this region dates back several decades, marked by intensive prospecting campaigns and extensive geological studies^23,24^. These efforts made it possible to discover and present several deposits, which contributed to local economic development. However, the exploitation of these resources continues to face significant challenges, particularly the difficult access conditions in this mountainous region and the environmental concerns associated with mining activities (Table 1; Fig. 1).

**Table 1:** Main deposits of the Eastern high atlas.

Deposit	Mineralization	Host rocks	Hosting strata	Ore minerals	Gangue and alteration minerals	Secondary alteration	Type of deposit	References
Merija	Cu	Sandstones and conglomerates (red beds)	Infra-Cenomanian	Chalcopyrite	Calcite	Malachite-Azurite-Fe-oxides	Sediment-hosted copper deposits	^25^
Jbel Skindis	Cu–Pb–Fe–Mn	Dolostones	Lower and Middle Lias	Chalcopyrite-Pyrite and Galena	Calcite/Dolomite	Fe-dolomite-Malachite-Covellite-hematite-geotheite and limonite		^11^
Bou Dahar	Zn–Pb–Ba	Reefal Limestone	Lower and Middle Lias	Sphalerite-Galena and Barite	Calcite	Celestine-Melnicovite-Malachite-Azurite-Aragonite-Tn-Clays and Fe-Mn (Hydro)oxides	Mississippi Valley-Type Deposits	^26,27^
Jbel El Klakh	Cu	Dolostones	Lias	Chalcopyrite-Pyrite-Galena and Sphalerite	Quartz and Dolomite	Malachite-Azurite-Chalcosine-Digenite-Covellite-Brochantite-Smithsonite-Anglesite-Clays and Fe-Mn (Hydro)oxides	Sediment-hosted copper deposits	^28^
Lhouanite	Cu	Dolostones	Jurassic	Chalcopyrite-Pyrite-Galena and Sphalerite	Quartz and Dolomite	Malachite-Azurite- Covellite-Smithsonite-Anglesite and Fe-Mn (Hydro)oxides	Sediment-hosted copper deposits	^28^
Bou Arfa	Mn–(Fe)	Dolostones	Sinemurian	Pyrolusite-Manganite- Hausmannite- Fe-oxides	Calcite/Dolomite	Calcite-Fe-oxides	Mississippi Valley-Type Deposits	^29^
Zelmou	Ba	Limstones and Schistes	Cambrian	Baryte			Vein-type barite deposits	^30^
Tamlalt-Menhouhou	Au, Cu, Ni, Co, As, Mo, Ag, ± Bi, Sb	Volcanic and volcano-sedimentary rocks	Neoproterozoic	Natif Au-Pyrite-Chalcopyrite-Molybdenite-Galena-Sphalerite-Baryte-Tennantite-tetrahedrite and Hematite	Quartz-Albite-Muscovite-Clays and Carbonates	Malachite-Covellite-Geothetite	Iron oxide copper-gold (IOCG) deposits/Shear zone related gold deposit	^31^

Recent studies have significantly advanced the understanding of mineralization processes in the EHA^32^ demonstrated the importance of structural lineaments in controlling mineralization in the region. Using Sentinel-1B and Envisat radar data, they identified major structural trends (N-S, NE-SW, and NW-SE) that are key in locating mineralized zones. These findings highlight the critical role of tectonic structures in the formation and distribution of Cu, Pb, and Zn deposits. Furthermore^33^, conducted a detailed petrographic and geochemical study of Jurassic-Cretaceous intrusive massifs in the EHA, focusing on gabbros and syenites. These intrusions, which formed in an intracontinental setting, are associated with a heterogeneous mantle source, enriched by plume-type melts. The study emphasized the role of magmatic and hydrothermal processes in the concentration of metals, with intrusions often surrounded by metasomatic zones and contact metamorphic aureoles, indicating the presence of hydrothermal fluids that contributed to mineralization.

In addition^34^, explored the genesis of Pb–Zn–(Ag–Fe–Cu) deposits in the northern Gangdese belt, showing that these deposits are linked to magmatic intrusions and hydrothermal fluids. The study demonstrated that the geochronology and isotopic analysis of lead and sulfur provided critical insights into the sources of ore-forming materials, confirming that magmatic-hydrothermal processes are fundamental in the formation of such deposits. These findings align with those of the EHA, where hydrothermal alteration and tectonic structures are key to identifying mineralized zones. Geochemically, previous research data from Pb–Zn deposits in the central and EHA, including Ali Ou Daoud, Sidi Belghite, Taabast, and Bou Dhar^29^, indicate similarities with Mississippi Valley Type (MVT) ore deposits^35^. Fluid inclusion studies show homogenization temperatures of 78–170 °C and salinities of 13–36 wt% NaCl equations^27,36,37^. The homogeneous Pb isotopic signatures suggest significant lead mobilization from the Paleozoic basement^38^. The age of Pb–Zn mineralization remains debated due to the lack of suitable minerals for radiometric dating and widespread post-mineral oxidation^39^.

Focusing more specifically on the study area, the Mougueur Inlier, a segment of the Hercynian chain from the Paleozoic era, reveals diverse mineral localities, including copper, iron, lead, and zinc. This region includes both active and abandoned mines, with mineralization occurring in the Paleozoic basement and Mesozoic cover. The deposits primarily occur as vein-type mineralization, consisting of carbonates^40^, late-stage quartz, and copper traces such as malachite and chalcopyrite. Notably, zinc carbonates within a massive Fe–Mn–Pb vein show significant oxidation, a characteristic emphasized in the Gourrama sheet, indicating their extensive reach.

This research addresses the lack of detailed exploration of the EHA by identifying mineralized zones in the Mougueur Inlier, High Atlas, Morocco, through the integration of remote sensing data and field observations. Utilizing the region’s semi-arid climate, sparse vegetation, and exposed bedrock, we employ multispectral satellite data to map potential mineralized areas by detecting hydrothermal alteration zones and structural features. A key objective is to explore the hydrothermal alteration zones and tectonic structures influencing mineralization, thereby increasing the likelihood of locating viable alteration zones for deeper exploitation.

## Geological setting

Our study focuses on the EHA region (Fig. 1A,B), specifically the Mougueur inlier, which is characterized by the outcrop of the Paleozoic basement. This area is situated in the northern part of the Atlas Domain. The terrains within the Mougueur inlier are outlined in Fig. 1B). The age assignment for each newly defined formation is established by comparing facies with the reference column in the Anti-Atlas, unless diagnostic fossils, particularly from the Silurian period, are present. Below, we provide concise descriptions of the identified formations, ordered from bottom to top.

The Middle Cambrian is manifested as outcrops of green schists, Paradoxides schists, and Tabanit Sandstone. These outcrops appear as elongated east-west bands, affected by regional faults. The Ordovician forms the majority of the inlier and is subdivided from the base to the top into green and red sandstone-pelitic formations (External Faijas), weakly differentiated schisto-sandstone facies, and a group of sandstones, quartzites, and microconglomeratic clays at the top. The Silurian is the only stage dated paleontologically (Graptolites). The outcrop of these facies is located in the Tijane depression in the form of tectonic lenses along the faults, subdivided into two main formations: graptolite clays topped by a turbiditic formation. The Devonian is represented by turbiditic formations succeeding the Silurian graptolite clays. At the western end of the Mougueur buttonhole and its western extension, Triassic terrains outcrop (C).

Tectonically, all Paleozoic terrains are affected by widespread Hercynian synschistous deformation associated with tangential tectonics with SSE to SE vergence, in a low-grade metamorphic context^41,42^. These tectono-metamorphic characteristics associate the Mougueur region with the Hercynian transition zone of the Mesitian domains. The Paleozoic basement assembly and its Meso-Cenozoic cover are affected by tectonic structures of the Atlas cycle, generally comprising two types^21,41,42^: extensional or transtensional events linked to the dislocations of Pangea and compressive events linked to the convergence of the Africa-Europe plates in the Cenozoic, leading to deformations in the Mesozoic and Cenozoic cover within the EHA domain^21^. From a mineralization perspective, the Mougueur anticline is distinguished by the presence of three mineralization forms exhibiting varied morphologies (cluster, stratiform, and vein) and diverse mineralogical compositions.

**Fig. 2:**
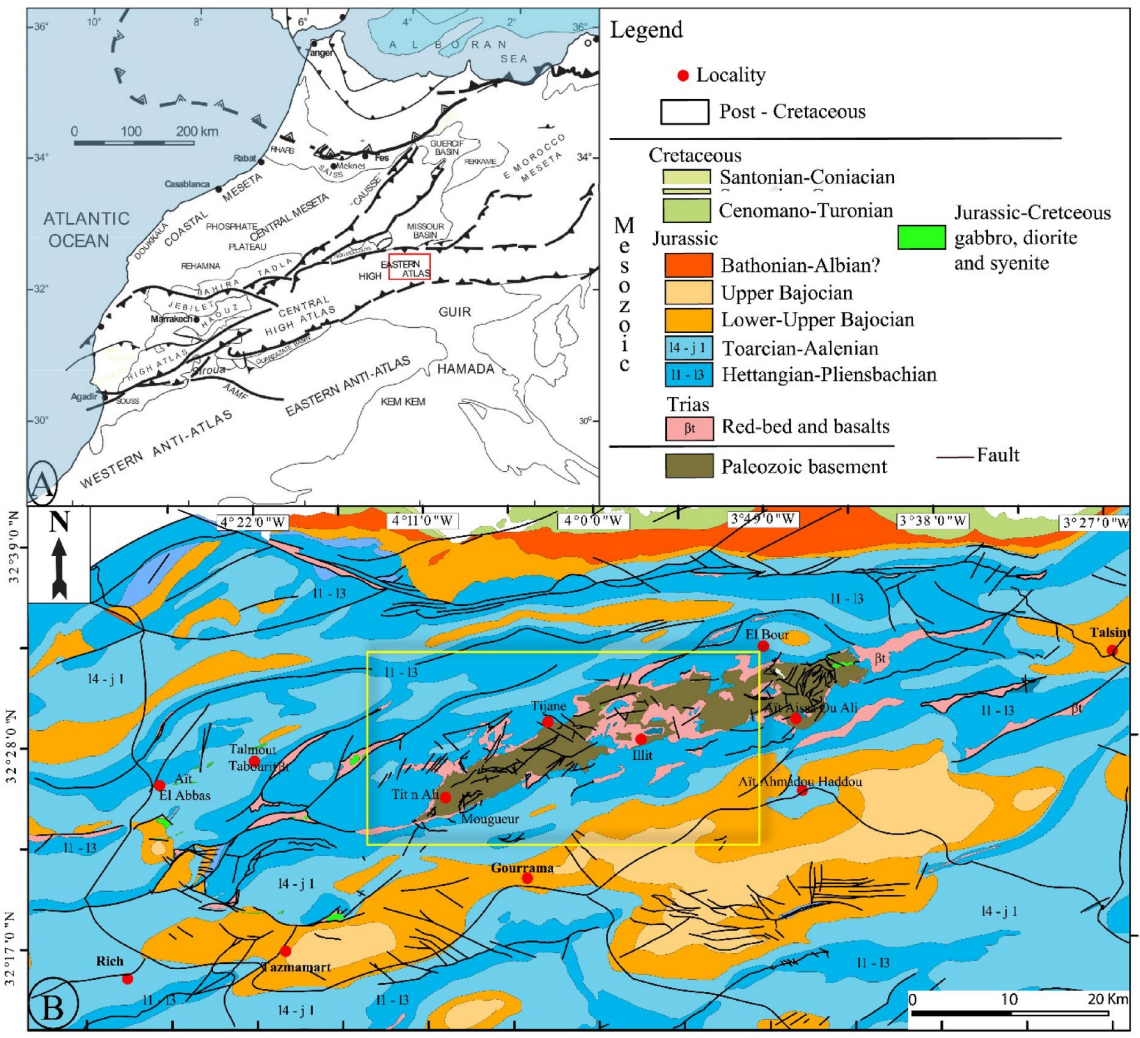
(**A**) Simplified structural map of north Morocco showing the localization of the Eastern High Atlas (from Frizon de Lamotte et al.^43^. (**B**) Simplified geological map of Eastern High Atlas Mougueur province (Allouban et al.^33^. Created by ArcGIS Desktop 10.8. https://www.esri.com/en-us/arcgis/products/arcgis-desktop/overview.

Additionally, the presence of intrusive rocks, including gabbro, syenite, and diorite, is already depicted in Fig. 2, where their distribution is clearly marked in light green. The geological setting (Fig. 2) describes these intrusions and their emplacement within Triassic-Jurassic folded sedimentary formations and the Paleozoic basement. This information provides further context regarding the intrusive rocks in the study area, illustrating their significance from a mineralization perspective.

## Materials and methods

### Data sources

In this investigation, the Advanced Spaceborne Thermal Emission and Reflection Radiometer (ASTER) images were employed. Characteristics of the utilized scene are summarized in Table 2, showing that it was captured under favorable weather conditions, featuring very low cloud cover. Launched in December 1999, ASTER is a sophisticated multispectral sensor equipped with 14 spectral bands, enabling it to capture a broad range of wavelengths compared to other multispectral sensors. These bands consist of three VNIR bands with a spatial resolution of 15 m, six SWIR bands with a spatial resolution of 30 m, and five TIR bands with a spatial resolution of 90 m, as detailed in Table 2. After preprocessing, which included reprojection of the acquired data to the Lambert Conformal Conic projection with the Mercich datum system, the current research emphasizes the consolidation and analysis of only the VNIR and SWIR bands due to their higher spatial resolution.

In this work, two types of remote sensing datasets were used: (a) an optical dataset, whose characteristics are given in Table 2, from Landsat 8 OLI scene acquired on April 3, 2019 from the website (https://earthexplorer.usgs.gov/) with scene cloud cover 3.13%. (b) ASTER scene acquired on August 17, 2005 from website (https://gbank.gsj.jp/madas/map/) with Scene cloud cover 1.00%.

**Table 2:** Summarized characteristics of the ASTER and Landsat-8 OLI.

OLI			ASTER		
Band	Central Wavelength (µm)	Pixel size (m)	Band	Central wavelength (µm)	Pixel size (m)
1	0.4430	30	1	0.5560	15
2	0.4826		2	0.6610	
3	0.5613		3 N	0.8070	
4	0.6546		3B	0.8070	
5	0.8646				
6	1.6090				
7	2.2010				
8	0.5917	15	4	1.6560	30
			5	2.1670	
			6	2.2090	
			7	2.2620	
			8	2.3360	
			9	2.4000	
9	1.3730	30	10	8.2910	90
			11	8.6340	
10	10.9000	100	12	9.0750	
			13	10.6570	
11	12.0000		14	11.3180	

### Methodology

**Fig. 3:**
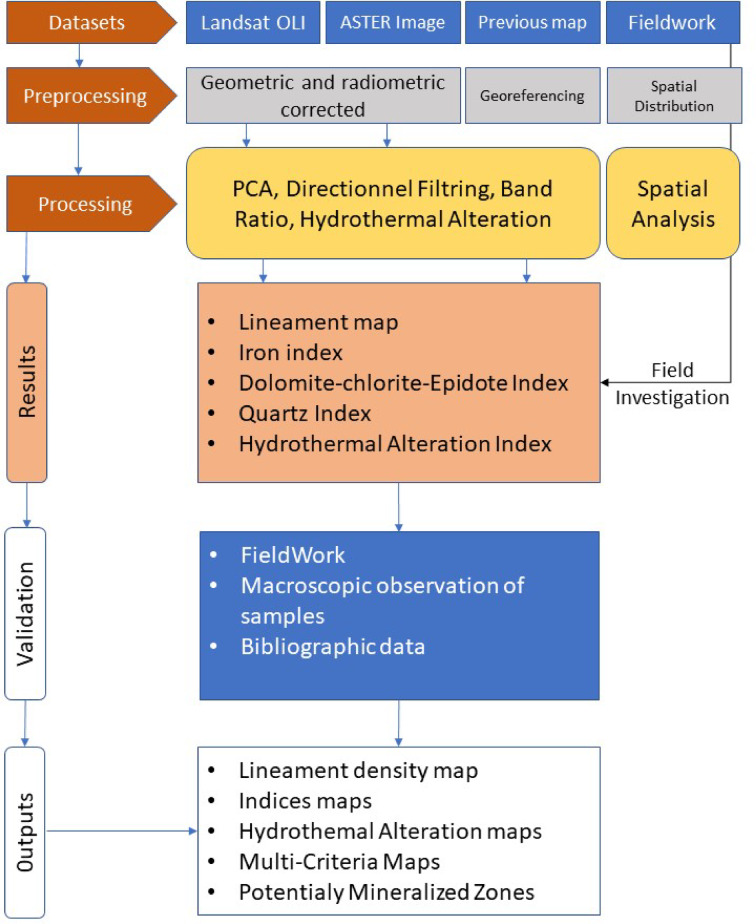
Flowchart methodology applied for the current research.

This study integrates advanced image processing techniques with a lineament extraction algorithm to delineate potential structural elements and hydrothermal alteration zones. The methodological workflow is summarized in Fig. 3 and described in detail below.

**Fig. 4:**
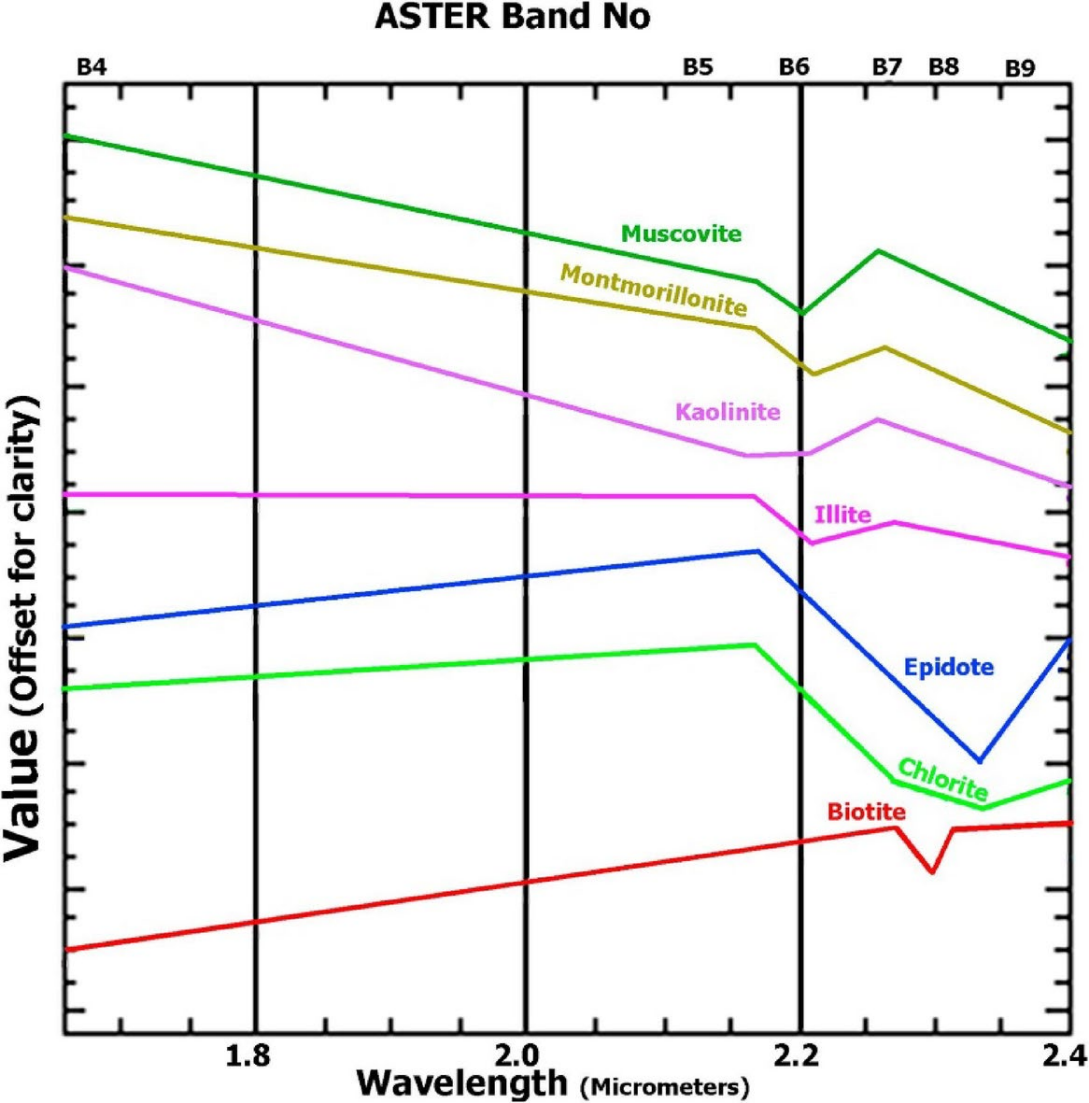
Laboratory reflectance spectra of selected end-member minerals extracted from the ASTER spectral library version 2.0^9,45^ that convolved to ASTER’s SWIR bands.

For lineament extraction, the Line Extraction module in Geomatica software was employed. This algorithm identifies linear features in satellite imagery and converts them into vector format. The process relies on six key parameters, including the filter radius, which smooths the image; the gradient threshold, which determines the minimum gradient required for detection; the length threshold, which sets the minimum length of detected lineaments; the line fitting error threshold, which controls the maximum allowable error for line fitting; the angular difference threshold, which defines the maximum angular deviation for linking segments; and the linking distance threshold, which specifies the maximum distance for connecting line segments. The extracted lineaments were subsequently imported into ArcMap and overlaid onto the 1:50,000-scale Gourrama geological map (Fig. 4). A systematic visual interpretation was conducted to exclude lineaments corresponding to geological contours, watercourses, and cliffs, retaining only those deemed structurally significant. This approach follows established methodologies as described in previous studies^10,12,15,16,44^.

To validate and further verify the detected lineaments, the generated lineament map was superimposed onto a 1:200000 topographic map. This step aimed to eliminate anthropogenic or irrelevant lineaments, such as roads, streams, and cliffs. The directional frequencies of lineaments were analyzed using rose diagrams, grouping lineaments into equal classes with a 10° amplitude.

Hydrothermal alteration mapping was performed to identify zones associated with base and precious metal deposits. Two complementary approaches were applied to ASTER data. First, the band ratio method was used to enhance spectral variations, enabling discrimination between different mineral and rock groups. Indices such as ferric iron (Band 4/Band 3), gossan (Band 4/Band 2), carbonate-chlorite-epidote ((Band 5 + Band 7)/Band 6), and quartz index (Band 11/(Band 10 + Band 12)) were calculated. Second, spectral analysis was conducted using laboratory reflectance spectra of key alteration minerals sourced from the ASTER spectral library version 2.0^46^. These spectra were convolved to match ASTER’s SWIR bands, ensuring compatibility between laboratory data and sensor capabilities. This approach provided critical insights into the spatial distribution and intensity of hydrothermal alteration zones, facilitating the identification of characteristic alteration minerals.

Hydrothermal alteration mapping was conducted using ASTER band ratios, following approaches validated in previous studies. Fieldwork and laboratory investigations were conducted in two phases. The first mission, carried out prior to image processing, involved geological reconnaissance and sample collection to identify metallic formations and associated hydrothermal alterations. Macroscopic analyses of the collected samples were performed in the laboratory to characterize mineralization and alteration. The second mission was organized after image processing and the synthesis of results, with the aim of validating the thematic map through direct observations and ground truthing in the study area.

This integrated methodology, combining remote sensing, field observations, and laboratory analyses, provides a robust framework for identifying structural controls and hydrothermal alteration zones. It represents a significant advancement in mineral exploration for the Eastern High Atlas region, offering valuable insights into the geological processes governing mineralization in this area.

Two field missions were conducted. The first took place before we began image processing and aimed to explore the study area, carrying out a geological synthesis of all metallic geological formations. Following this mission, we performed macroscopic analyses of the samples in the laboratory to observe the various mineralizations and associated alterations. The second mission was organized after processing and synthesizing the results to validate the final thematic map.

## Results

## Structural analysis

**Fig. 5:**
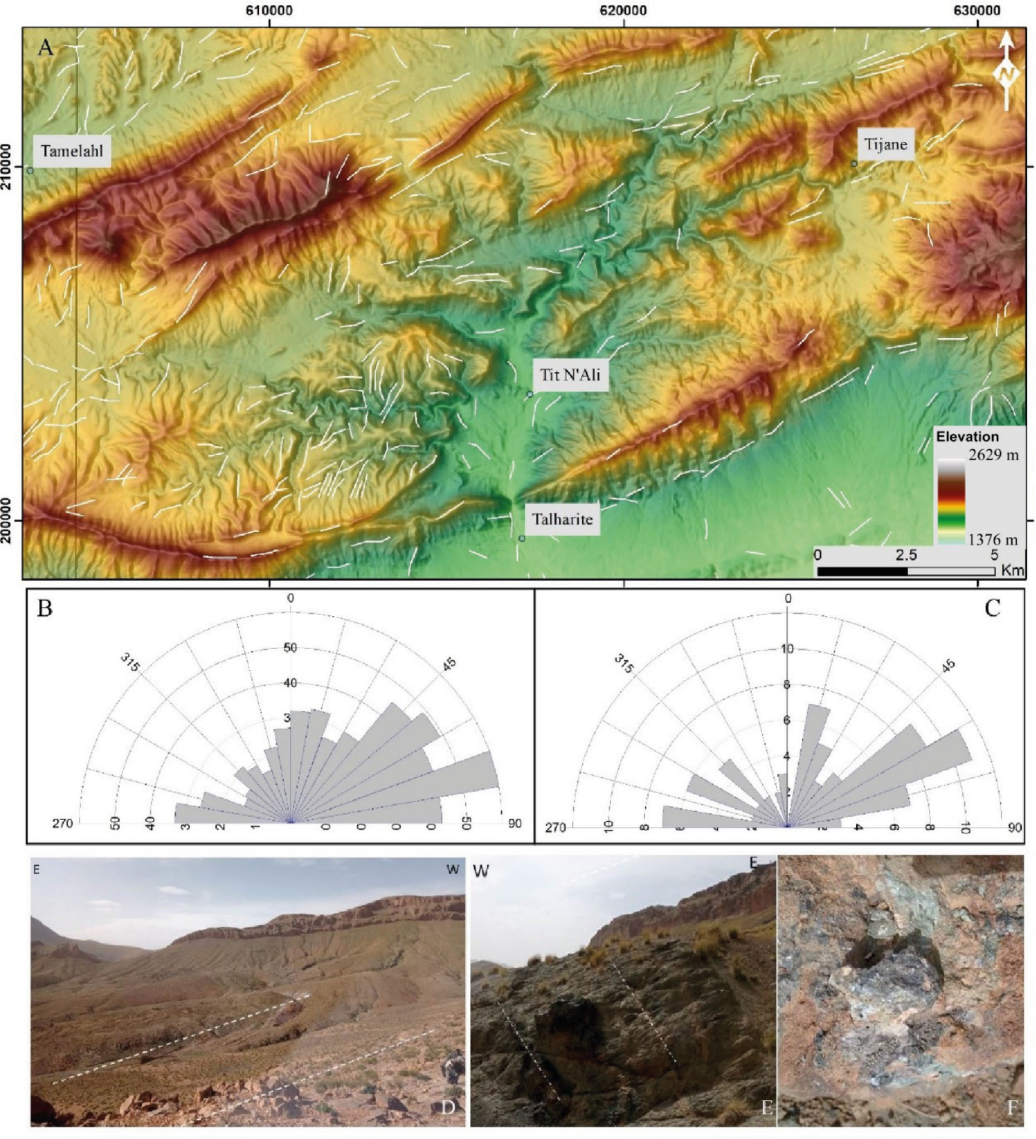
(**A**) Map of Lineaments showing their distributions and orientations; (**B**) Rose diagram of lineament orientations with an interval of 10° (**C**) Rose diagram of Faults (**D**,**E**) faults observed in field Photo at position P26 in Fig. 11 (**F**) Fault-related copper mineralization Photo at position P27 in Fig. 11. The figure (5a) was created by SmartSketch v. 4.0 software; https://smartsketch.software.informer.com/4.0/, ArcGIS Desktop 10.8. https://www.esri.com/en-us/arcgis/products/arcgis-desktop/overview, and ENVI v. 5.6.2. software; (https://www.l3harrisgeospatial.com/Software-Technology/ENVI).

**Fig. 6:**
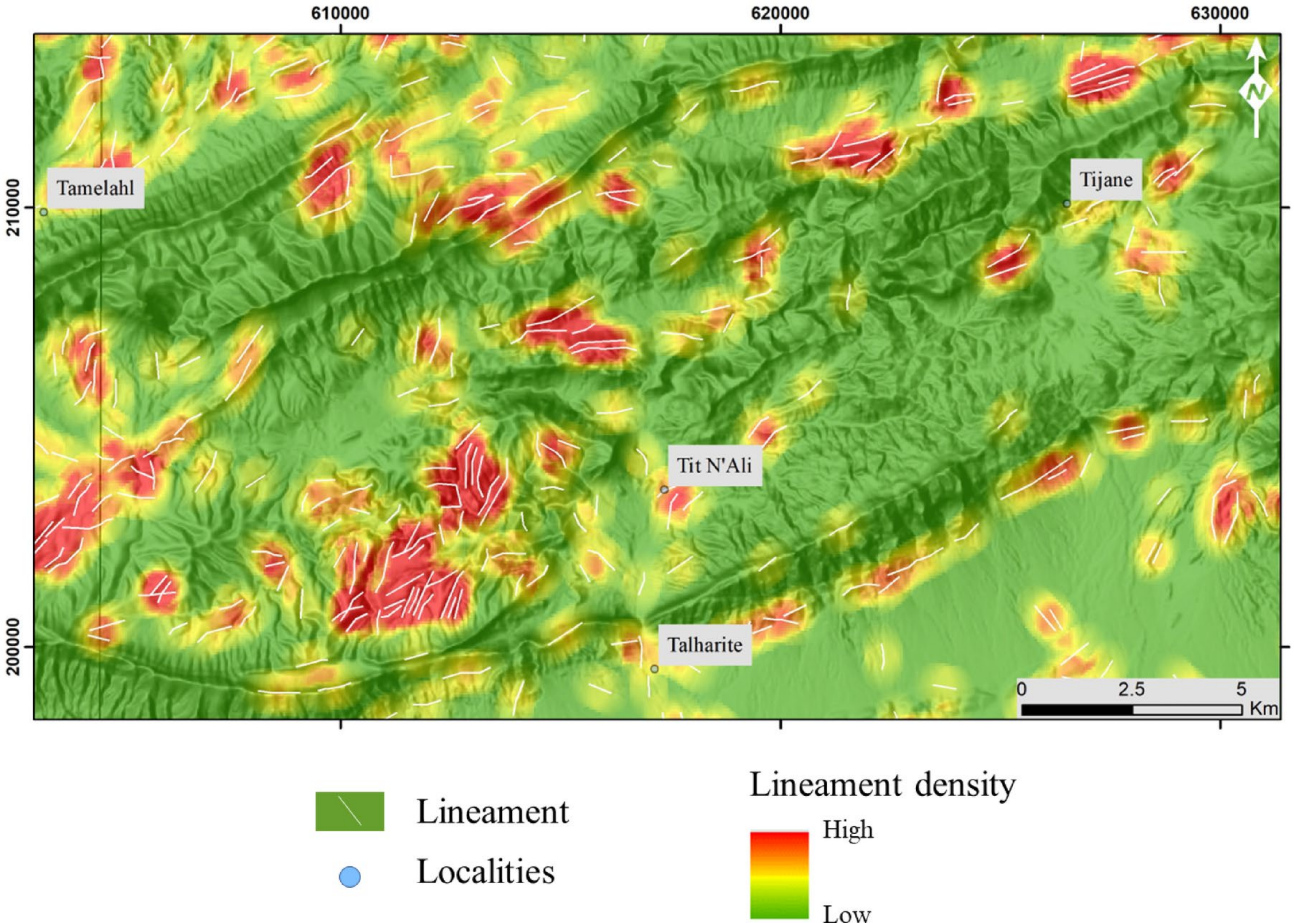
Lineaments density map for the study area. The figure was created by SmartSketch v. 4.0 software; https://smartsketch.software.informer.com/4.0/, ArcGIS Desktop 10.8. https://www.esri.com/en-us/arcgis/products/arcgis-desktop/overview, and ENVI v. 5.6.2. software; (https://www.l3harrisgeospatial.com/Software-Technology/ENVI).

Our lineament extraction analysis revealed that the area is highly fractured (Fig. 5). The geometric characteristics of the lineament network and the dominatnt regional orientations wer examined using statistical analyses. The rose diagram of lineament directions reveals prevailing directions ranging from NE-SW to SE-NW, with the ENE-WSW (N80) direction dominating, accounting for 42% of the lineaments (Figs. 5B,C). The dotted lines are based on the observation of brecciation, which is used to identify faults in the field. These directions are verified with field observations of fault-related copper mineralization. (Figs. 5D–F). Through various processing operations, a composite map of fracturing was derived, showing a total of 1528 lineaments. Analysis of the results reveals a preferential distribution of lineaments in the Tit N’ali, Tamelahl, and western Talharite area, as well as at the Tijane location. These lineaments are organized into corridors with orientations predominantly NE-SW and NNE-SSW, particularly to the north of Tit N’ali and Tijane as shown over the lineament density map (Fig. 6). This analysis highlights the efficiency of the utilized Landsat data in extracting linear features. The extracted lineaments, as shown in Figs. 5 and 6, have been validated through our field observations.

### Hydrothermal alteration mapping

Due to the wealth of SWIR bands in ASTER compared to Landsat OLI, the hydrothermal alteration mapping was performed utilizing ASTER data. Band ratios derived from ASTER data proved to be valuable for the qualitative identification of alteration minerals, including kaolinite, alunite, and iron oxides, as indicated by mineral indices^47,48^. Special attention was given to iron oxides, dolomite-chlorite-epidote, and quartz, as these are the most important indicators of mineralization within the studied terrain, based on our field observations and previous research.

#### Band ratio for Iron detection

**Fig. 7:**
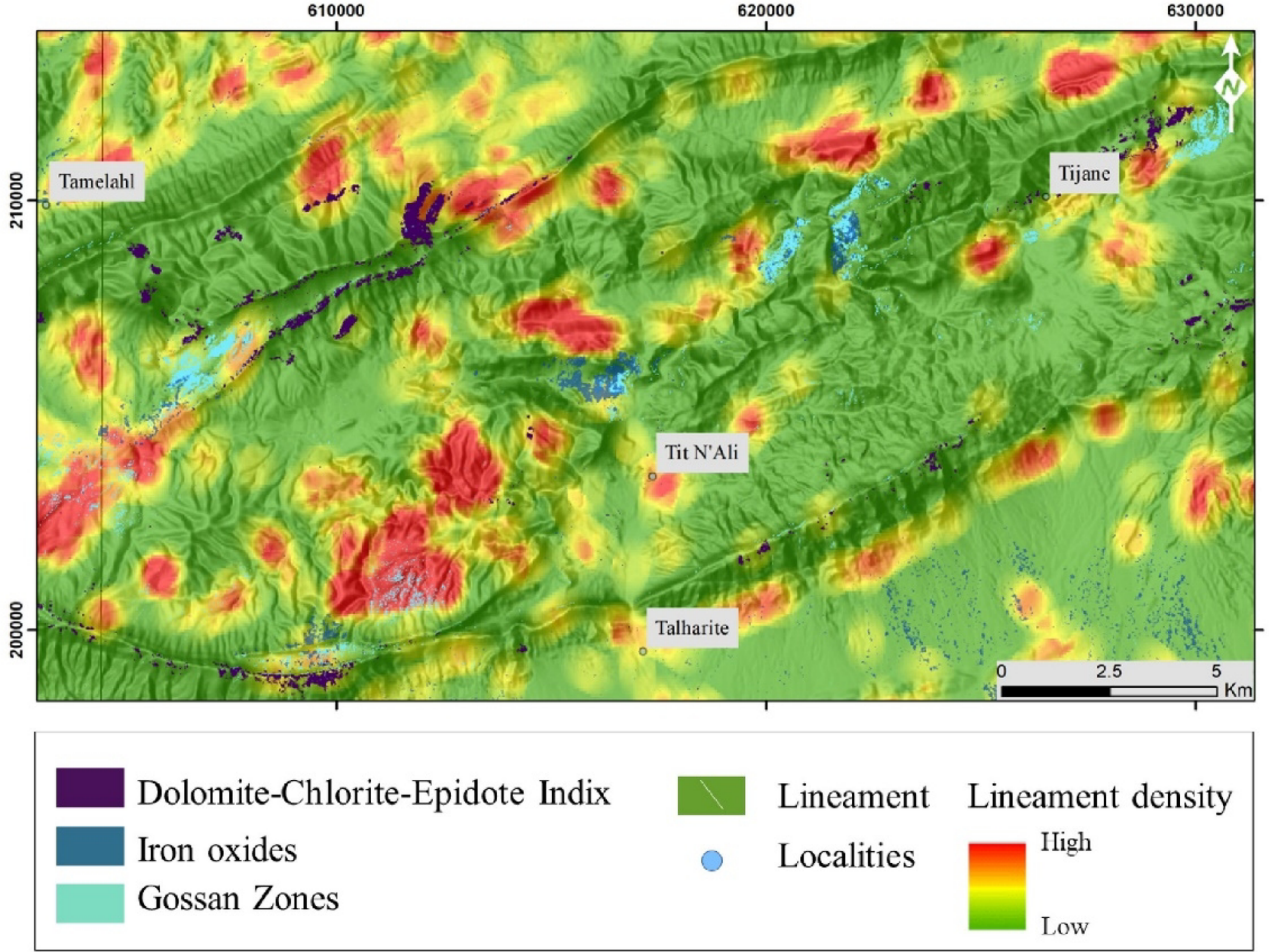
Spatial overlay analysis of the primary hydrothermal alteration indices—dolomite-chlorite-epidote ((b5 + b7)/b6), iron content (4/2), and gossan (3/2)—superimposed on lineament density map. The figure was created by ArcGIS Desktop 10.8. https://www.esri.com/en-us/arcgis/products/arcgis-desktop/overview, and ENVI v. 5.6.2. software; (https://www.l3harrisgeospatial.com/Software-Technology/ENVI).

The band ratio (b4/b2)^49^ is utilized for detecting gossan hats, which represent the superficial part of the deposit, characterized by high oxygenation and hydration due to atmospheric and biospheric interactions (Jébrak and Marcoux, 2008). The distribution map of the Gossan index shows that its signature aligns in corridors and appears either as concentrated spots or scattered points (Fig. 7).

#### Dolomite-chlorite-epidote index

The ASTER band math of ((b5 + b7)/b6) is used to map locations rich in Dolomite-chlorite-epidote (van der Meer et al., 2012). The signature is well developed in the areas of Tijane, north of Tit N’ali, west and east of Talharite (Fig. 7). The development of these signatures aligns closely with fault orientations ranging from NE-SW to E-W and NW-SE.

#### Quartz index

**Fig. 8:**
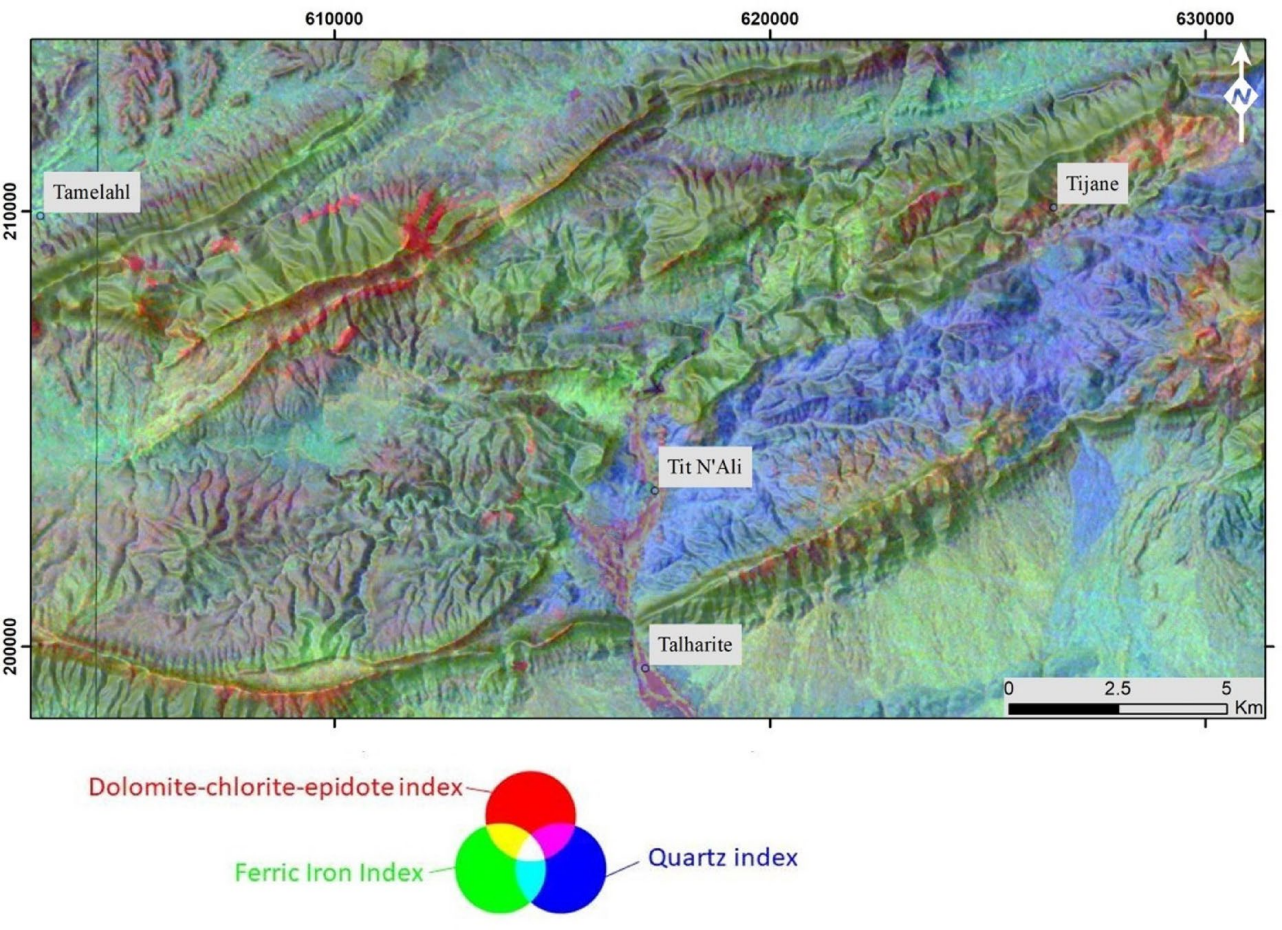
Hydrothermal alteration mapping through a combination of band ratios highlighting dolomite-chlorite-epidote (R), Iron content (G), and quartz (B). The figure was created by ArcGIS Desktop 10.8. https://www.esri.com/en-us/arcgis/products/arcgis-desktop/overview, and ENVI v. 5.6.2. software; (https://www.l3harrisgeospatial.com/Software-Technology/ENVI).

The distribution map of the quartz index shows that the signature of the latter is aligned with an E-W direction in the heart of the inlier, these places are characterized by the presence of quartz veins with traces of copper minerals (Malachite and chalcopyrites) and iron (Figs. 7 and 8). It is noteworthy that certain nineralization localities correlate with the mapped faults (Fig. 7). Furthermore, the alignment of these results with the iron and copper localities identified in the Gourrama sheet supports the hypothesis regarding the effectiveness of this band ratio for mapping potentially mineralized zones.

#### Distribution patterns and intersections of the studied hydrothermal alterations

This process involves creating a composite image by combining three band ratios of iron, dolomite-chlorite-epidote, and quartz (Fig. 8). This approach enhances the visibility of their overlap, providing insights into the presence of these indices within each geological formation.

**Fig. 9:**
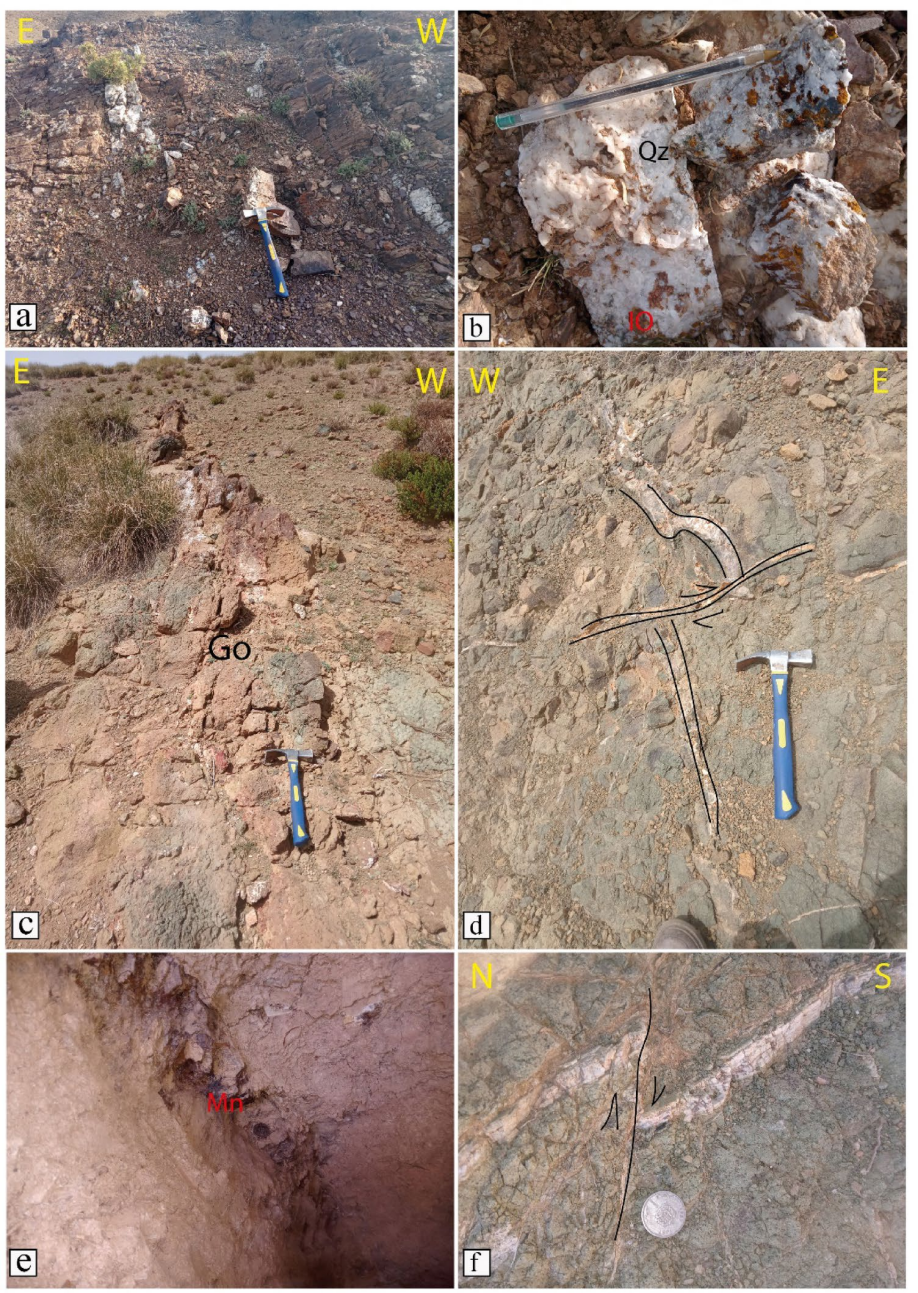
(**a**) Quartz vein associated with a tectonic structure. (**b**) Quartz (Qz) with iron oxides. (**c**) Gossan (Go) in an altered outcrop. (**d**) calcite veins deformed by tectonic structures. (**e**) Manganese (Mn) mineralization in a rock cavity. (**f**) Calcite vein crosscut by a tectonic fracture.

In Fig. 8, the light blue areas correspond to copper-iron mapping localities, highlighting a connection to ferric oxidation associated with the development of quartz veins within the sandstone-pelite shales of the Paleozoic basement, as further illustrated in Fig. 9. This copper-iron mineralization can be regarded as a promising metallotect. Yellow zones correspond to regions known for Pb–Zn mineralization, characterized by associations with iron oxidation minerals and carbonates, notably lacking quartz. The purple areas indicate the presence of both silica and chlorite, primarily observed in the Triassic basalt-gabbro formations. Field observations suggest this coloration results from the hydrothermal alteration of biotite into chlorite in gabbroic zones affected by NE-trending faults. These targets also show disseminated copper mineralization within the fractured basalt.

Dark green areas indicate a prevalence of ferric oxidation in the Triassic basalts, particularly in regions known for the occurrence of iron veins embedded in this Triassic formation. Light brown color is associated with the dolomitization of the Upper Lias limestone. This coloration lacks iron indices but features a silica signature, possibly attributed to detrital quartz intercalations in the quartzite layers of this Liassic formation (Figs. 8 and 9).

### Hydrothermalism component

The RGB hydrothermal composite, based on the band ratios b4/b6, b2/b1, and b3/b2 (Fig. 8), effectively highlights altered zones and illustrates their distribution across the study area. These altered zones are shown in light red, aligning with the mapped shear corridors. These corridors coincide with areas of dolomite-chlorite-epidote development as shown by our field investigations. However, this signature is not prominent in the Cenozoic formations, except for isolated traces west of Tamelahl and northwest of Talharite.

## Field verification and data investigations

**Fig. 10:**
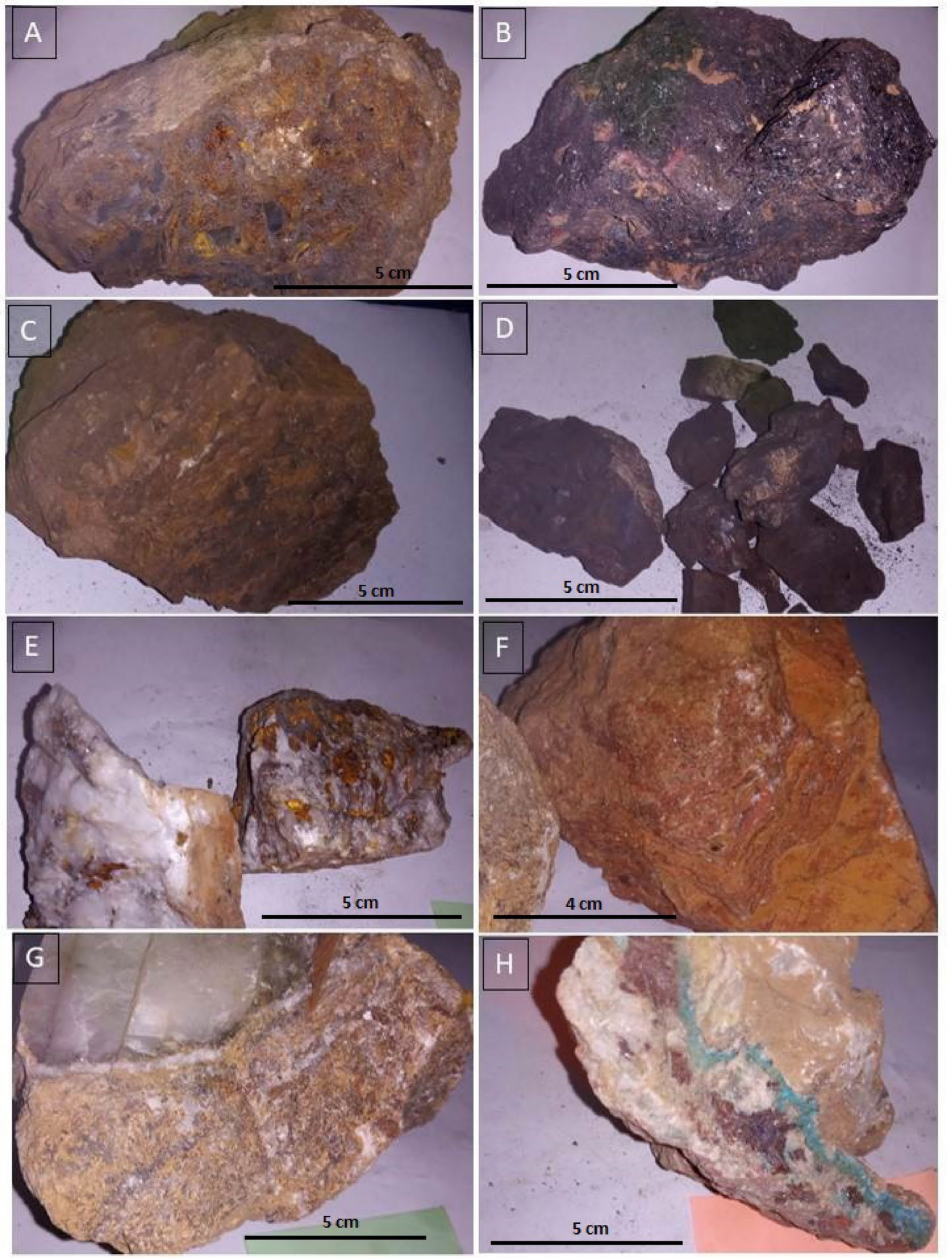
(**A**)–(**C**) smithsonite associated with P21 iron oxides. (**B**) sphalerite P29. (**D**) Manganese ore P28. (**E**) Quartz with iron oxide inclusions. (**F**) Smithsonite with P21 iron oxidation. (**G**) Zn smithsonite ore with P21 calcite crystals. (**H**) Origin of malachite associated with iron oxides P26. P refers to the field station point.

**Fig. 11:**
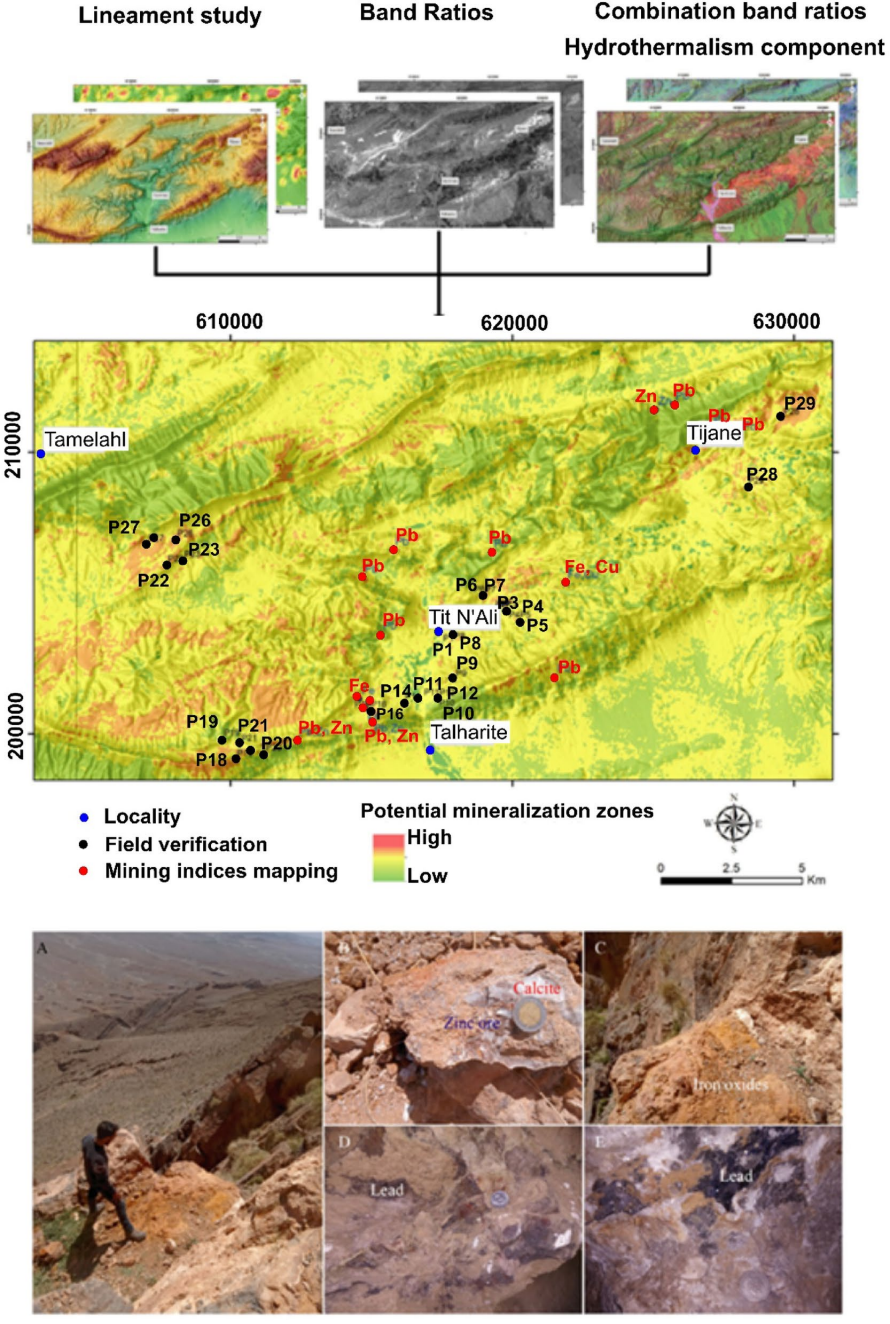
The mineralization potential map shows strong correspondence, with points mainly in yellow to red zones (moderate to high potential), aligning with old mining localities and verified field sites (shown as dots). The field photographs depict: (**A**–**C**) iron oxides associated with zinc mineralization, and (**C**–**E**) lead mineralization, highlighting other promising areas. The figure was created by ArcGIS Desktop 10.8. https://www.esri.com/en-us/arcgis/products/arcgis-desktop/overview, and ENVI v. 5.6.2. software; (https://www.l3harrisgeospatial.com/Software-Technology/ENVI).

Extensive fieldwork was conducted in the study area to validate our findings. Our field observations revealed various types of quartz and calcite veins, along with prominent gossans, highlighting hydrothermal activity in the region (Fig. 9). Additionally, alteration zones were observed along the walls of mineralized veins, marked by the presence of clay and iron minerals such as limonite and goethite (Fig. 10). These alteration minerals serve as key indicators in satellite imagery. Our remote sensing results have been verified using field data (the distribution of the main field stations is shown in Fig. 11), known mineralized zones, and previous studies (Fig. 11). Fieldwork was carried out to verify hydrothermal alteration zones and highly dissected areas (Figs. 5 and 6, and 7), and displayed in Fig. 11 to manifest the power of the ASTER and Landsat OLI combination by targeting areas of actual hydrothermal alteration within the study area. A great harmony between the detected alterations and our field observations were noted (Figs. 5D–F and 10A,H). Our field investigations revealed different types of hydrothermal alteration minerals, demonstrating the role of current research in deciphering the hydrothermal alteration model of the study area.

Our analysis of the structural findings indicates that the lineaments are concentrated preferentially in the heart of the SW part of the inlier and are organized along large fault corridors and aligned with the quartz and iron oxide indices (Fig. 7). The overlay of lineaments, iron oxide, and quartz indices on a single map reveal a strong correlation (Fig. 7). Notably, areas outside the core of the inlier exhibit a pronounced iron oxide signature, likely resulting from extensive oxidation of the Triassic basalts and along fractures within the zinc carbonate deposits (Fig. 7). In contrast, within the Paleozoic formations, these indices align with the major fault corridors trending NE to ENE, as well as along E-W trending quartz veins, reflecting structural control in the mineralization zones identified in the index maps.

In terms of mineralization, the quartz and iron oxide signatures align with the mapped iron and copper localities (Figs. 7, 8 and 11, GPS points P2, P3, and P28, Fig. 7). The zinc and lead carbonate localities overlap with the iron oxide signatures, forming linear patterns (Figs. 7, 8 and 11 GPS points P21, P22, and P28, Fig. 7A,B). The copper and iron vein mineralization in the basement are hosted within the schist-sandstone series, intrusions, and gabbroic dikes. These mineralized structures manifest as vein swarms with multi-metric extensions oriented ENE-WSW, WNW-ESE, and E-W, occurring in karst formations and fractures.

Field observations reveal a mineralogical assemblage comprising galena, pyrite, and chalcopyrite. Additionally, supergene alteration of these sulfides has resulted in the formation of secondary minerals such as cerussite, malachite, azurite, and iron oxides (Fig. 10E,H). Alteration zones were also observed along the walls of the mineralized veins, characterized by the presence of clay and iron minerals, including limonite and goethite (Fig. 10A,C). These alteration minerals serve as key indicators for the detection of mineralized zones in satellite imagery.

## Multi-criteria analysis

The objective of this section is to refine data interpretation through the integration of simultaneous cross-referencing and geospatial overlay analysis, incorporating both satellite-derived structural and hydrothermal data alongside field observations. This approach, widely adopted in previous studies, has demonstrated its effectiveness in mineral deposit targeting (Shebl et al., 2021). These processing techniques aim to synergize diverse datasets, uncovering new geospatial relationships (Scanvic, 1993). In this study, results from satellite image processing—specifically lineament and mineral index analyses—are compared with cartographic surveys of mineralized veins to establish prospecting criteria for identifying potential exploration zones.

The overlaid map resulting from this integration (Fig. 11) illustrates the alignment of regions with high lineament density (indicating strong fracturing) and gossan showings with the locations of known mineralized veins in the inlier. Mapping these veins onto the colored hydrothermal compound map demonstrates a strong correlation with zones of hydrothermal alteration (Fig. 11). Notably, these veins either follow the main shear corridors or exhibit an oblique orientation to them.

This analysis reveals that mineralized zones correspond to areas characterized by extensive fracturing and alteration, containing clay minerals and iron oxides. The significance of faults becomes evident as major conduits for mineralizing fluids within these zones, with fluid circulation facilitated by the extensive fracturing.

Favorable mineralized zones are mainly controlled by a structural regime and associated with one or more types of alteration. ASTER and Landsat 8 datasets were integrated to describe the structural control regime and the common alteration zones to finalize the results by constructing a mineralization potential map for the study area. The elements resulting from the calculation of band ratios and the color composition as well as the lineaments extracted have been harmonized. The results showed that the area was affected by several tectonic trends classified NW-SE, NE-SW, NS and E-W according to their importance.

The NE-SE structural trend is the dominant trend common to mineral deposits; However, NE-SW to E-W was represented locally. These trends seem to reasonably coincide with previous studies. Considerable correspondence is also evident between lineament density, heat maps from remote sensing. Initial discrimination of the altered hydrothermal zones was carried out by remote sensing in argillic, advanced argillic, phyllic, and propylitic alteration, as well as manifested zones enriched in gossan, ferric oxide, and carbonate-chlorite-epidote. Towards a weighted superimposed analysis, all results were manipulated as layers in a GIS environment and each layer (lineaments, density heatmaps, and type of alteration) received the same value (weight) without any preference because they have approximately the same importance in defining the target. Therefore, highly weighted values ​​are assumed to be the collective representation of structural complexity and alteration zones, which strongly indicate favorable mineralized sites, and vice versa. The results are displayed in the form of a mineralization potential map. This is confirmed by the two field checks (Fig. 9) and the previously mapped mining localities. This, in turn, manifests the effectiveness role of combined datasets used in robust recommendations for areas of mineralization. This integrated approach suggests five areas with high potential for the installation of minerals and located SW of Tamlahl, in the Tijane region, NE and SW of Tit N’Ali and in the SW part of the study area. three areas are already confirmed by mining activity.

**Fig. 12:**
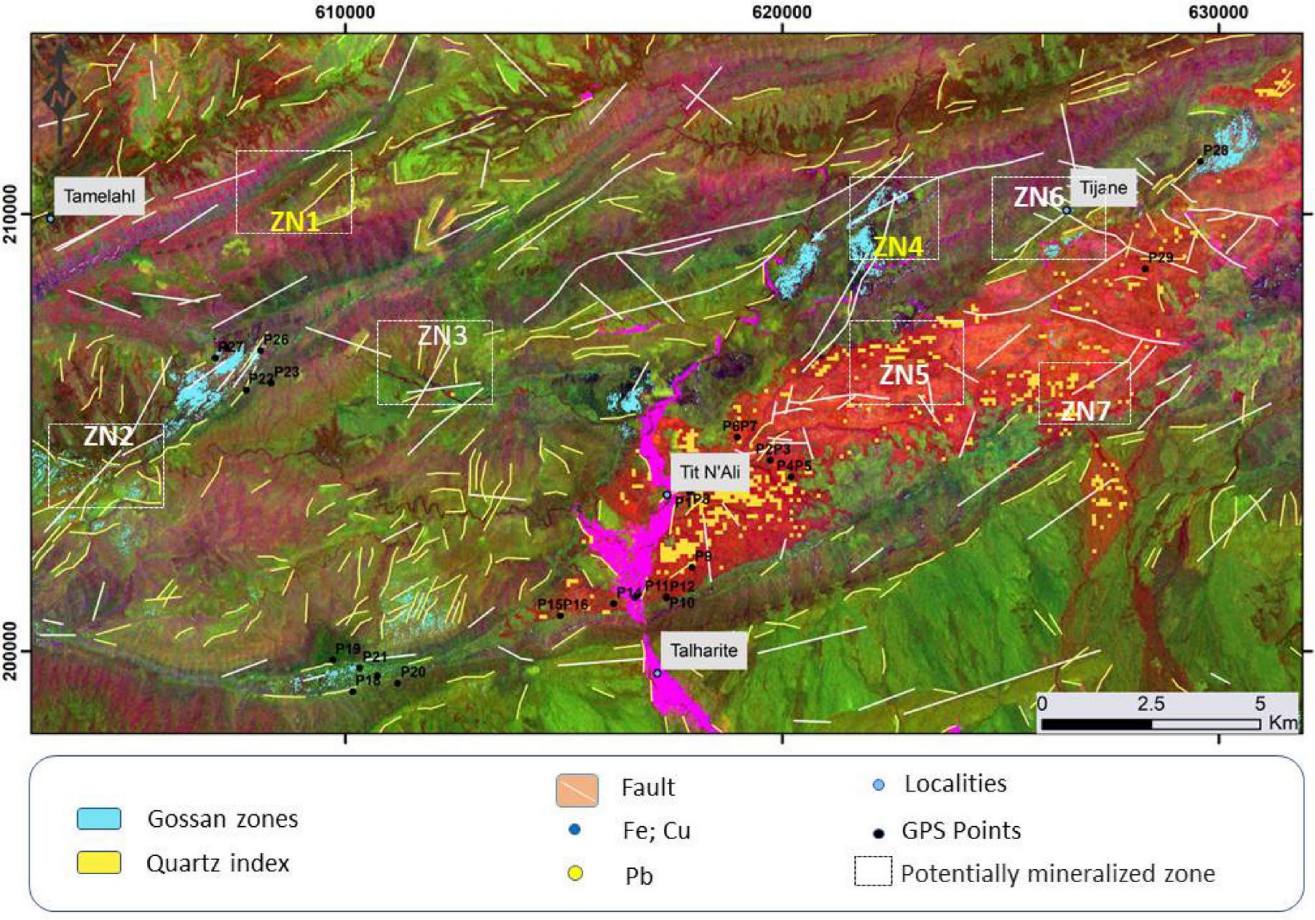
Spatial overlay of lineament and mineral alteration maps, combined with field observations and known mineralizations, displayed over the ASTER RGB composite (5/7, 3/1, 4/3), highlighting prospective mineralized zones (ZN1–ZN6). The figure ArcGIS Desktop 10.8. https://www.esri.com/en-us/arcgis/products/arcgis-desktop/overview, and ENVI v. 5.6.2. software; (https://www.l3harrisgeospatial.com/Software-Technology/ENVI).

## Discussion

Automated extraction of lineaments from Landsat 8 OLI images revealed a high density of lineaments in specific localities such as Tit N’ali, Tijan, Talharit, and Tamelahl. These areas correspond to intense fracturing in the NE–SW and NW–SE shear zones (Fig. 5). These results are consistent with previous studies showing that lineament density is a key indicator of potentially mineralized areas (Pour & Hashim, 2015). Alteration minerals such as Gossan, dolomite-chlorite-epidote, and hydrothermal minerals, extracted from ASTER images, were closely associated with the main fault zones (Tit N’ali, Tijane, and Tamelahl) (Fig. 5). These findings corroborate the works of Sabins^50^ and Carranza^51^, which demonstrated that fault zones facilitate the circulation of hydrothermal fluids, leading to alteration and deposition of mineralizations.

Alteration minerals, such as Gossan, dolomite-chlorite-epidote, and hydrothermal minerals, identified through ASTER imagery, were closely associated with the main fault zones, including Tit N’ali, Tijane, and Tamelahl. Hydrothermal indices aligned with fault orientations further support the idea that these structures control the circulation of mineralizing fluids. This is consistent with the work of Bentahar et al.^32,52^, who demonstrated that fault zones, particularly those oriented NE-SW, are crucial in facilitating the movement of hydrothermal fluids, leading to alteration and deposition of mineralizations. Their study, based on radar data from Sentinel-1B, corroborates our findings showing that fault-controlled zones are central to mineralization in the region.

The superimposition of alteration zones and mineralized veins on the lineament density map revealed a good alignment with the most fractured and altered areas. This indicates that shear zones and associated fractures containing hydrothermal alteration minerals are the most promising targets for the exploration of Pb–Zn and Cu–Fe mineralized veins in the Inlier of Mougueur. These results align with the conclusions of Pour and Hashim^53^, who used similar techniques to identify mineralized area.

Our research identified several areas as new potential zones that have not been mined before. For instance, the NE–SW corridors of Tit N’ali, NW–SE of Tamelahl, and east of Tijane have been identified as new potential areas with no prior mining activity (Figs. 7, 11 and 12). Additionally, the SE extension of the exploited veins at Tit N’ali and the eastern extension of Tijane have been recognized as targets for potential mining exploration. These discoveries are consistent with the works of Carranza^51^, who demonstrated that integrating remote sensing data with machine learning models can reveal previously undetected mining exploration targets.

The work of Allouban et al.^33^ on magmatic intrusions in the EHA also provides important insights into the formation of metallic deposits in the region. Their geochemical study of Jurassic-Cretaceous intrusive massifs, composed of gabbros and syenites, revealed that these intrusions, formed in an intracontinental setting, are associated with a heterogeneous mantle source enriched by plume-type melts. They highlighted the role of magmatic and hydrothermal processes in metal concentration, particularly in areas surrounding intrusions where hydrothermal fluids facilitated mineralization. These findings align reasonably with our observations in the Mougueur Inlier, where magmatic intrusions and contact metamorphic zones play a similar role in the formation of deposits.

Furthermore,^34^ confirmed the crucial role of magmatic and hydrothermal processes in the formation of Pb–Zn and other metal deposits in the northern Gangdese belt (located in the southern Tibetan Plateau, is a well-known polymetallic belt, which hosts important deposits of copper, gold, molybdenum, iron, zinc, lead, silver). Their study showed that magmatic intrusions and hydrothermal fluids are responsible for the concentration of metals in Pb–Zn deposits. The geochronological and isotopic analysis of lead and sulfur provided critical insights into the sources of ore-forming materials, confirming that these magmatic-hydrothermal processes are fundamental in the formation of such deposits. These conclusions are also applicable to the EHA, where similar processes play a key role in the localization of mineral deposits.

Notwithstanding the usefulness of remote sensing’s pivotal role in our research, it also has limitations. A primary drawback is the spatial resolution of satellite data, which may fail to capture minor geological features essential for detailed mineral exploration. Additionally, atmospheric conditions and vegetation cover can affect spectral signatures, potentially leading to inaccuracies in detecting mineral deposits in vegetated terrains. Nevertheless, the benefits of remote sensing for our current contribution are considerable, as it allows for the efficient exploration of vast and remote areas that would be difficult and costly to assess using traditional methods. It also enhances the discrimination of specific mineral signatures through techniques like spectral band ratios and principal component analysis (PCA), making it a valuable tool in early-stage exploration. Additionally, it pinpoints several new zones as potential exploration targets. The success of this approach in the Inlier of Mougueur serves as a model for future prospecting initiatives in similar deposits throughout the Eastern High Atlas and comparable regions. Future work will focus on integrating high-resolution hyperspectral data with GIS, alongside geological, geophysical, and geochemical data. This comprehensive methodology will explore the application of machine learning algorithms for effective multi-criteria analysis, thereby enhancing the potential for discovering new mineralized areas (Rodriguez-Galiano et al., 2015; Cracknell & Reading, 2014).

## Conclusion

Remote sensing and GIS tools, complemented by field observations, played a crucial role in comprehensively mapping geological formations, alteration zones, and structural discontinuities. Leveraging Landsat 8 OLI and ASTER data, this study highlighted the potentiality of zinc–lead ± copper vein mineralization in the Mougueur Inlier. The current research concluded the following:

- The automated extraction of lineaments from Landsat 8 OLI images facilitated the mapping of key structural discontinuities in the basement, exhibiting strong correlation with on-site surveys. This technique delineated the anticlinorium structure of schist formations and identified major faults-oriented NE–SW, N–S, and ESE–WNW. The high lineament density in specific localities, including Tit N’ali, Tijan, Talharit, and Tamelahl, corresponded to intense fracturing in NE–SW and NW–SE shear zones.
- Our remote sensing findings and field investigations highlighted the distribution of Gossan, dolomite-chlorite-epidote, and hydrothermal minerals, extracted from ASTER images, and showed that they were closely associated with primary fault zones (Tit N’ali, Tijane, and Tamelahl). These fault zones facilitated the circulation of hydrothermal fluids, leading to alteration and mineralization deposition in the Mougueur Inlier.
- Overlaying alteration zones and mineralized veins onto the lineament density map revealed a clear alignment with the most fractured and altered areas. These zones, identified as structural discontinuities containing hydrothermal alteration minerals, represent promising targets for Pb–Zn and Cu–Fe mineral exploration in the Mougueur Inlier.
- The study identified new high-potential mining zones with no prior activity, including the NE–SW corridor of Tit N’ali, the NW–SE corridor of Tamelahl, and the eastern part of Tijane. Additionally, the SE extension of veins exploited at Tit N’ali and the eastern extension of Tijane were recognized as strategic targets for further exploration.
- These findings provide valuable insights for decision-makers to support the development of the mining sector in the Eastern High Atlas.

## Data Availability

The datasets used and/or analyzed during the current study are available from the corresponding author upon reasonable request.
